# Financing and health system capacity for precision medicine in Asia: a six country landscape analysis

**DOI:** 10.1093/haschl/qxag238

**Published:** 2026-09-01

**Authors:** Adena Li Tyin Peh, Yot Teerawattananon, Jeanette Xue Yan Yuen, Nordin Charafi, Dicky Levenus Tahapary, Jiahua Leng, Alec Morton, Sheyu Li, Woo Jin Kim, Bin Alwi Zilfalil, Hidayati Husainy Hasbullah, Nusara Satproedprai, Shao-Tzu Li, Joanne Yuen Yie Ngeow, Yah Ru Juang, Yue Zhang, Minji Hong, Wenjia Chen

**Affiliations:** Saw Swee Hock School of Public Health, National University of Singapore, Singapore 117549, Singapore; Health Intervention and Technology Assessment Program (HITAP), Department of Health, Ministry of Public Health, Nonthaburi 11000, Thailand; Department of Cancer Genetics Service, National Cancer Centre Singapore, Singapore 168583, Singapore; Illumina Pte Ltd, Singapore 138667, Singapore; Metabolic, Cardiovascular, and Aging Research Cluster, The Indonesian Medical Education and Research Institute, Faculty of Medicine Universitas Indonesia, Jakarta 10430, Indonesia; Key Laboratory of Carcinogenesis and Translational Research (MOE/Beijing), Gastrointestinal Center Unit III, Peking University Cancer Hospital & Institute, Beijing 100142, China; Saw Swee Hock School of Public Health, National University of Singapore, Singapore 117549, Singapore; Department of Endocrinology and Metabolism, West China Hospital, Sichuan University, Chengdu 610041, China; Department of Internal Medicine, Kangwon National University Hospital, Kangwon National University, Chuncheon 24289, South Korea; Human Genome Center, School of Medical Sciences, Health Campus, Universiti Sains Malaysia, 16150 Kubang Kerian, Kelantan, Malaysia; Human Genome Center, School of Medical Sciences, Health Campus, Universiti Sains Malaysia, 16150 Kubang Kerian, Kelantan, Malaysia; Eastern Economic Corridor Office of Thailand (EECO), Bangkok 10500, Thailand; Department of Cancer Genetics Service, National Cancer Centre Singapore, Singapore 168583, Singapore; Department of Cancer Genetics Service, National Cancer Centre Singapore, Singapore 168583, Singapore; Saw Swee Hock School of Public Health, National University of Singapore, Singapore 117549, Singapore; Saw Swee Hock School of Public Health, National University of Singapore, Singapore 117549, Singapore; Saw Swee Hock School of Public Health, National University of Singapore, Singapore 117549, Singapore; Saw Swee Hock School of Public Health, National University of Singapore, Singapore 117549, Singapore

**Keywords:** precision medicine, pharmacogenomics, genomic medicine, reimbursement policy, health technology assessment, Asia, implementation science

## Abstract

**Background:**

Precision medicine (PM) adoption is accelerating across Asia, but implementation remains uneven due to differences in financing, infrastructure, governance, and health-system readiness.

**Objectives:**

To examine how six Asian countries (Singapore, South Korea, China, Malaysia, Thailand, and Indonesia) adopt, finance, and integrate PM technologies, and identify common implementation patterns and challenges.

**Methods:**

A landscape review of peer-reviewed literature, government publications, and HTA reports (2010-2026) was conducted, supplemented by stakeholder consultations. PM applications were grouped into public health screening (hereditary breast and ovarian cancer [HBOC] and familial hypercholesterolemia [FH] cascade testing), next-generation sequencing (NGS) applications (rare diseases, oncology, pharmacogenomics), and AI-enabled PM. Evidence was synthesized across access, awareness, reimbursement, and implementation.

**Results:**

Public health genomic screening demonstrated the highest implementation readiness, followed by precision oncology, while rare disease diagnostics remained infrastructure-dependent and pharmacogenomics and AI-enabled PM platforms were at earlier stages. Four readiness profiles emerged: highly aligned systems; reimbursement-constrained systems with strong governance and infrastructure; systems strengthening governance, public financing and infrastructure; and strategy-led systems expanding implementation through pilot programs and referral centers.

**Conclusions:**

PM implementation across Asia remains heterogeneous. The identified readiness profiles highlight governance, financing, and infrastructure priorities for sustainable and equitable PM diffusion.

Key Points

- Reimbursement emerged as one of the most consistently observed factors associated with implementation maturity across countries: Countries with more aligned financing and national insurance coverage demonstrate broader and more routine adoption, while reliance on out-of-pocket or mixed financing models limits scalability despite technological availability.
- A triad of strategic financing, infrastructure, and governance underpins sustainable adoption: Effective implementation depends on the alignment of reimbursement pathways, digital and laboratory capacity, and coordinated national strategies, highlighting that progress in any single domain alone is insufficient.
- Persistent gaps in evidence, workforce, and awareness constrain equitable access: Limited real-world and cost-effectiveness evidence, shortages in genetic counseling capacity, and uneven awareness continue to hinder scale-up, underscoring the need for strengthened HTA systems, workforce investment, and regional collaboration.

## Introduction

Precision medicine (PM) integrates genomic, environmental, and clinical information to tailor disease prevention, diagnosis, and treatment to individuals or specific population groups. By moving beyond a traditional “one-size-fits-all” care model, PM has the potential to improve the effectiveness of healthcare delivery. Its successful implementation depends on coordinated financing, governance, infrastructure, workforce capacity, and data systems. Globally, PM has demonstrated potential to improve clinical outcomes and cost-effectiveness, particularly in cancer, rare diseases, and cardiovascular conditions.^1-5^

In Asia, several countries have prioritized PM within national health strategies. Singapore's National Precision Medicine program supports population genomics, while South Korea has integrated genomic medicine into national biohealth strategies supported by HTA and reimbursement mechanisms.^6^ China has invested significantly in genomics, data integration, and artificial intelligence (AI), though implementation varies across regions.^7^ Malaysia, Thailand, and Indonesia have also incorporated PM into national health strategies,^8^ though some initiatives remain pilot-based.^9^ These efforts reflect growing momentum with differences in health-system capacity and financing.

PM has progressed from single-gene testing to multi-gene panels (eg, hereditary breast and ovarian cancer [HBOC]) and next-generation sequencing (NGS), expanding applications in rare disease diagnosis, infectious disease pathogen genomics, precision oncology, and pharmacogenomics.^10,11^ Emerging AI-enabled precision health platforms further support genomic interpretation and personalized care.^12,13^ Among PM applications, HBOC testing and familial hypercholesterolemia (FH) cascade screening have demonstrated value as population-level prevention strategies and are publicly or partially reimbursed in several Western countries (eg, the United Kingdom, United States, and parts of Europe).^14-17^ However, implementation remains heterogeneous across Asian health systems.^18-21^

Although PM is traditionally associated with individualized diagnosis and treatment, its scope increasingly extends to population genomic screening and AI-enabled applications. This review examines three categories of PM: (1) public health risk screening programs (HBOC testing, FH cascade screening); (2) NGS-enabled precision and companion diagnostics for rare diseases, oncology, pharmacogenomics, and infectious diseases; and (3) AI-enabled PM platforms that support clinical decision-making and continuous system learning. Collectively, these applications represent a continuum from population-level risk identification and prevention to precision diagnosis and treatment selection, and ultimately to adaptive precision health systems. Examining them collectively provides insights into the governance, financing, infrastructure, workforce, and evidence-generation capacities required for PM implementation.

Chong et al. (2018) reviewed PM readiness in Southeast Asia (2004-2017), focusing on early applications such as newborn screening, oncology therapies, and limited multigene sequencing. The review highlighted substantial variation in governance, access, and financing, with implementation largely early-stage and champion-driven. However, newer PM applications and key countries such as China and South Korea were not included. More recently, regional initiatives such as the HTAsiaLink Precision Health Transformative Technology Assessment Initiative (PHAT-TAI) evaluated HTA readiness across 12 Asia-Pacific territories, highlighting substantial variations in evidence assessment capacity and decision-making structures.^22^ However, broader implementation barriers affecting equitable access to PM remain insufficiently understood. Building on these earlier assessments, this study examines a broader range of countries and recent and emerging PM applications that collectively represent an evolving precision health ecosystem. By examining applications across prevention, diagnosis, treatment, and adaptive learning, we identify implementation priorities for developing integrated, adaptive precision health systems across diverse health-system contexts.

As part of the PHAT-TAI initiative, this landscape analysis examines three PM categories across six Asian countries, focusing on national strategy, governance, infrastructure, data systems, reimbursement, regulation, and patient and social aspects. The findings inform subsequent stakeholder engagement and consensus-building activities to support sustainable PM implementation.

## Methods

### Study design

A landscape review following PRISMA-ScR guidelines (Supplementary File) was conducted to assess adoption, financing, and integration of advanced PM technologies across six Asian countries (Singapore, Malaysia, Indonesia, Thailand, South Korea, and China) selected under the PHAT-TAI initiative, building on the HTAsiaLink Precision Health Transformative Technology Assessment Initiative (PHAT-TAI) led by NUS and HTAsiaLink (2025).^22^ These countries were selected to represent diversity in health-system capacities and stages of PM development across East and Southeast Asia. Literature searches were conducted in PubMed, Scopus, EMBASE, and Web of Science, supplemented by gray literature (HTA reports and policy documents identified through targeted website searches).

### Scope of PM technologies

PM technologies were grouped into three categories:

1. **Public health risk screening programs:** HBOC genetic testing; FH cascade screening
2. **Precision diagnostics and companion diagnostics:** NGS-enabled diagnostics and treatment selection (rare diseases, oncology), and predominantly reactive pharmacogenomic testing
3. **AI-integrated precision health system:** AI-enabled PM platforms for genomic analysis and personalized treatment

A structured search (last date search: 28^th^ February 2026) used MeSH and free-text terms across three domains: (1) PM interventions, (2) financing and integration, and (3) geographic context. The full search strategy is provided in the Supplementary File. Consistent with PRISMA-ScR guidance, formal critical appraisal was not conducted because the objective was to map the breadth of PM implementation rather than evaluate intervention effectiveness.

### Inclusion and exclusion criteria

Studies were included if they addressed at least one PM application, reported system-level outcomes (eg, reimbursement, access, implementation), pertained to the six countries, and were published in English (2010 to 2026). Studies focusing solely on preclinical research, outside-scope technologies, conference abstracts, or opinion pieces were excluded.

### Study selection

**Figure 1.**
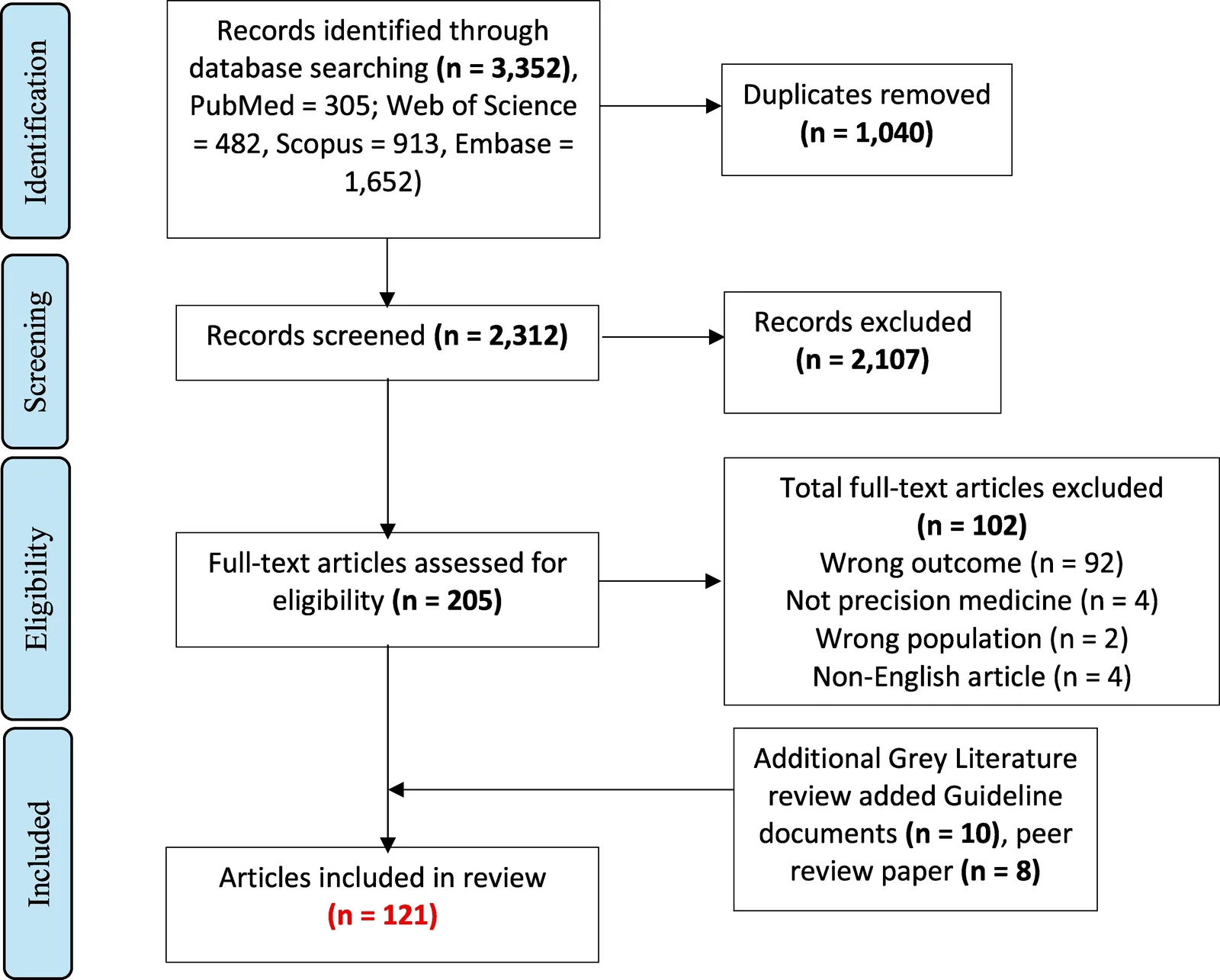
PRISMA flowchart displaying the process of identification and selection of included publications.

Database searches identified 3352 records; after removing 1040 duplicates, 2312 were screened. Of these, 205 underwent full-text review, and 103 were included. Additional gray literature (10 guidelines, 8 articles) yielded a total of 121 records (Figure 1). Data extracted included PM type (HBOC, FH, NGS, AI), reimbursement mechanisms, system integration, and outcomes related to access, awareness, and implementation, informing the comparative analyses.

### Analytical framework and data extraction

The Population, Intervention, Reimbursement, Outcome (PIRO) framework guided data extraction for cross-country comparisons of PM implementation.^23^ Building on Chong et al. (2018), implementation maturity was assessed qualitatively across six dimensions: routine care availability, initiating stakeholders, financing and reimbursement, monitoring and evaluation, healthcare integration, and digital infrastructure. Classifications were derived through iterative synthesis of the available evidence, with qualitative classification criteria provided in Table S1.

Given the exploratory nature of the review, a formal inter-rater reliability assessment was not performed. Therefore, maturity and awareness classifications should be interpreted as indicative rather than directly comparable quantitative measures. Following comparative synthesis, recurring themes informed the development of the Precision Medicine Implementation Triad, an interpretive synthesis framework that integrates financing, infrastructure, and governance to facilitate comparative policy analysis across health-system contexts.

### Stakeholder consultation

Stakeholder consultations were conducted to review and contextualize the landscape findings. Participants were recruited through purposive and snowball sampling as part of the subsequent PHAT-TAI interview and Delphi studies on PM reimbursement barriers and enablers in Asia. Prior to these studies, participants reviewed the country-specific landscape findings and provided feedback regarding their accuracy, completeness, and contextual relevance. Feedback was used to validate, triangulate, and contextualize the findings of the landscape review.

### Evidence synthesis

**Figure 2.**
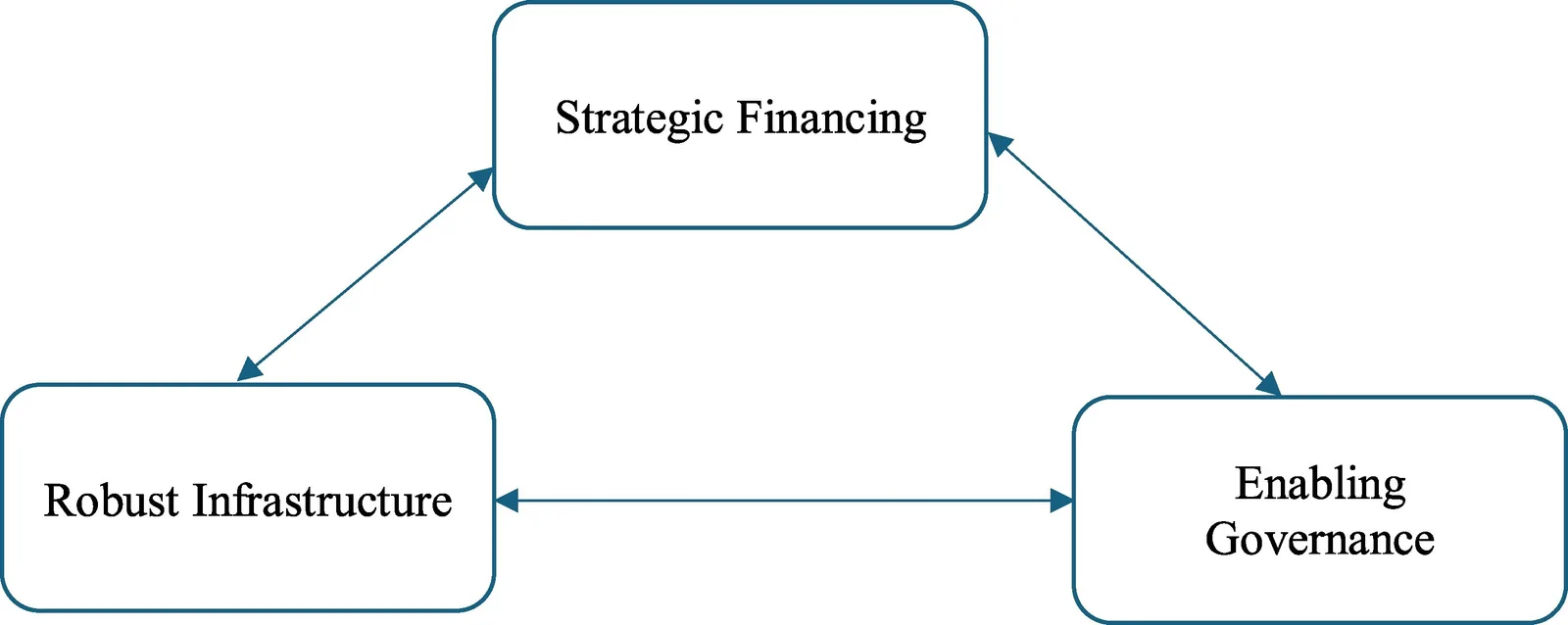
Precision Medicine Implementation Triad showing that sustainable PM implementation requires alignment across all three domains.

To synthesize findings, we developed the Precision Medicine Implementation Triad (Figure 2), an interpretive synthesis framework comprising three interconnected domains: 1) financing, 2) infrastructure, and 3) governance. Rather than replacing broader frameworks such as the WHO Health System Building Blocks or the Consolidated Framework for Implementation Research (CFIR), the Triad integrates these domains to facilitate comparative policy analysis, enabling the synthesis of country readiness profiles and identification of implementation priorities across diverse health-system contexts.

## Results

### Country-specific PM capacity

Table 1 presents an overview of PM system capacity across six countries. Additional details are provided in Tables S2-S7, with abbreviations in the Appendix.

**Table 1.** System-level enablers of precision medicine maturity in East Asian and Southeast Asian countries.

Country	Governance & national strategy	Financing & reimbursement	Digital infrastructure	Workforce capacity
Singapore	Strong national PM strategy (MOH, A*STAR, PRECISE)	Mixed financing; subsidies for FH & HBOC, most NGS self-pay	Advanced NGEMR with genomic integration	Strong workforce; limited genetic counselling capacity
South Korea	Strong national genomics strategy (MOHW)	Broad NHI coverage for HBOC and tumor NGS	Advanced national datasets and AI platforms	Strong workforce; some counselling gaps
Thailand	National genomics strategy (MOPH, HITAP)	UHC coverage for HBOC and selected PGx; limited NGS reimbursement	Moderate digital integration	Growing capacity with regional disparities
China	National oversight with tertiary-center leadership	Predominantly self-pay with selective municipal pilots	Strong tertiary-center infrastructure; fragmented nationally	Large but uneven workforce
Malaysia	Emerging national PM strategy	Mixed funding; selective public support, substantial self-pay	Expanding digital infrastructure	Limited but growing workforce
Indonesia	Emerging national PM strategy	Minimal public financing; largely self-pay or research-funded	Expanding digital systems (Satu Sehat)	Limited specialist capacity

### System-level enablers of PM maturity

PM maturity varied substantially across countries due to differences in governance (Table S2), financing (Table S3), and digital infrastructure (Tables S4 and S5). South Korea and Singapore demonstrated more advanced systems, supported by strong central coordination and national genomics strategies.^24-30^ In Singapore, the 10-year National Precision Medicine (NPM) program (launched in 2017) underpins initiatives such as SG100K and RapidSeq, with PRECISE supporting clinical implementation and integration into NGEMR. Clinical pilots have enabled translation into practice, including subsidized HBOC and FH testing.^24-28^ Similarly, South Korea's platforms, including K-MASTER, KOSMOS, and the Korean National DNA (K-DNA) program, support integration into clinical care.^31,32^

Thailand showed growing maturity through UHC-driven implementation and a national genomics program, though capacity remains concentrated in major centers^33-36^ China has achieved rapid technological expansion through tertiary hospitals and national investment,^37-41^ but implementation remains uneven due to fragmented data systems.^40,42,43^ In Malaysia, national initiatives such as MyGenome have been introduced, alongside ongoing efforts to develop a coordinated NPM strategy involving the Ministry of Health (MOH), Institute for Medical Research (IMR), MOSTI, and Ministry of Higher Education (MOHE).^44^ In Indonesia, the Genomic Sovereignty Initiative (GSI) and FH-focused program led by Cipto Mangunkusumo National Referral Hospital (RSCM)^45^ have supported the development of local genetic panels and early PM services.^46^

### Financing emerged as a consistent factor associated with PM adoption

As summarized in Table S3, systems with established reimbursement pathways show broader and more routine integration. In Thailand, HBOC testing and selected pharmacogenomic markers are reimbursed under UHC and the Civil Servant Medical Benefit Scheme (CSMBS), while South Korea's National Health Insurance (NHI) covers HBOC testing and tumor NGS panels, supporting routine oncology use^29,30,47-50^ In contrast, PM in Singapore is funded through institutional budgets, research grants, and out-of-pocket payments^51^ (Table S6) for financing and structures by country. While FH genetic testing is subsidized (up to 70%) with MediSave co-payment, most NGS applications remain uncovered.^24,25^ China largely operates under self-pay models with limited municipal reimbursement,^52^ Malaysia supports selected PM services through government-funded initiatives in public tertiary centers,^53^ while Indonesia has more out-of-pocket payment with lesser public reimbursement^54-60^ This reflects that where reimbursement or public financing mechanisms align with clinical pathways, PM is integrated into routine care; otherwise, implementation remains fragmented.

### Public health risk screening programmes: limited scaling despite supporting evidence

Cost-effectiveness evidence supports FH cascade screening and HBOC testing in selected settings, although transferability across countries remains uncertain. Singapore has a more developed FH cascade screening pathway, supported by subsidies, structured referral pathways, and primary care integration.^51,61^ Thailand and South Korea show more partial implementation, often limited to specialist-led services or pilot programs^33-36^ In China, FH testing is typically linked to treatment eligibility rather than systematic cascade screening^37-41^ Malaysia's MyHEBAT initiative has improved detection but lacks a standardized pathway,^62^ while Indonesia's BGSi^63^ program has strengthened national genomics infrastructure through an integrated, hub-based approach^54-60^ Overall, these findings suggest that even well-established interventions require coordinated financing, governance, and delivery to achieve population-level scale.

### Precision diagnostics: availability outpaces system integration

For rare disease diagnostics, implementation differed substantially across countries (Table S7). South Korea has established national programs supported by NHI coverage,^64,65^ while Singapore delivers rare disease NGS through structured programs like BRIDGES and the Undiagnosed Disease Program.^66^ Thailand is expanding access through national genomics hubs, though services remain concentrated in tertiary centers.^8,35^ China has scaled genomics through large initiatives (eg, UPWARDS), analyzing over 94 000 individuals, but access varies regionally.^67-69^ In contrast, Malaysia is transitioning from research-led access towards early clinical integration, with a national PM strategy currently under development^54-60^ In oncology, tumor NGS is widely implemented in tertiary centers across countries. South Korea demonstrates the strongest integration through reimbursement under NHI,^29,70,71^ while China has rapidly expanded access through commercial providers aligned with national priorities.^42,72^ Elsewhere, access remains dependent on private provision or institutional funding.^73^ Overall, technological capability has advanced faster than financing and system integration.

### Pharmacogenomics: evidence-constrained rather than technology-limited

Pharmacogenomics shows a distinct implementation pattern (Table S7). Thailand leads regionally, with reimbursement for selected drug–gene pairs under UHC and strong guideline integration.^33-36,74^ China has achieved broader uptake across oncology and infectious diseases, though implementation remains uneven.^75^ Singapore and South Korea have adopted pharmacogenomic testing in selected clinical areas,^76,77^ while Malaysia remains in a transitional stage, with limited testing offered in tertiary centers on a self-pay basis.^78,79^ Unlike other PM applications, the primary constraint is not infrastructure but insufficient clinical and economic evidence to support widespread reimbursement and standardization.^8,76^

### Digital infrastructure and healthcare integration as enablers of scale

Digital maturity (Table S4) and healthcare integration (Table S7) underpin the scalability of PM. South Korea demonstrates a more advanced ecosystem, with integrated national datasets enabling linkage of genomic and clinical data for longitudinal monitoring^31,32,80^ while Singapore shows strong integration through NGEMR and national genomics platforms.^24-28^ Both countries have embedded PM across clinical pathways, supported by multidisciplinary workflows and national infrastructure.^24,26-29,50,51,70,71,81^ China has developed advanced genomic systems within tertiary centers but lacks consistent national integration,^37-42^ while Thailand's digital systems support monitoring through ICD-based coding and reimbursement tracking, particularly for pharmacogenomics^33-36^ Malaysia is progressing towards early clinical integration through selected public referral centers,^82^ whereas Indonesia has more limited routine integration into healthcare systems^46,54-60,83-85^

Across countries, workforce shortages, particularly in genetic counseling and bioinformatics, further constrain implementation and scale.^37-39,83,84,86^

Next, findings were analyzed through the lenses of access (Table S8), awareness (Table S9) and implementation (Tables S10 and S11) of the six key PM types across each country.

Access to PM varies across technologies, reflecting differences in financing arrangements, health-system readiness, and implementation priorities identified above (Table 2).

**Table 2.** Implementation of precision medicine by technology and country.

Precision medicine type	Singapore	South Korea	Thailand	China	Malaysia	Indonesia
HBOC testing	Public access; upcoming subsidies; high awareness	NHI-reimbursed BRCA; high awareness	UHC-reimbursed (high-risk); increasing awareness	Tertiary centers; self-pay	Public referral centers; government-funded	Private sector; self-pay
FH cascade screening	National program; subsidized	Lipid clinics; partial reimbursement	Pilot-based	Linked to treatment eligibility	Academic pilots	Limited availability
Rare disease diagnosis	National programs; strong implementation	NHI-covered; national network	Genomics hubs; expanding	Large-scale but uneven	National referral center; funded for eligible cases	Emerging genomics initiative
Precision oncology	Routine tumor NGS; Multi-cancer early detection (MCED) research programs	NHI-reimbursed; integrated care	Tertiary-center access; expanding	Commercially driven; uneven access	Selected funded programmes	Selected oncology companion diagnostics reimbursed
Pharmacogenomics	Targeted use; pilot-based	Selective reimbursement; established uptake	UHC-reimbursed markers; advanced implementation	Broad but uneven adoption	Limited tertiary-center use	Limited clinical implementation
AI-enabled PM	Research-driven pilots	National platforms; most advanced	Early clinical adoption	Rapid tertiary-center deployment	Emerging pilots	Emerging national initiatives

### Public health risk screening programs: relationship between financing and scale

For population-level interventions, access follows financing structures. South Korea, Thailand, and Malaysia provide public coverage for HBOC testing in higher-risk populations, supporting wider uptake.^77,87^ In contrast, China and Indonesia have a mix of self-pay financing, limiting access to tertiary and urban settings, while Singapore is transitioning towards subsidized financing following HTA evaluation.^18,42,56,84,88^ FH cascade screening remains largely pilot-based across the region, with Singapore demonstrating the most developed model through structured referral pathways and subsidies,^61^ while other countries rely on more research-driven approaches.^62^ These findings suggest that even cost-effective interventions remain underutilized without coordinated financing and delivery systems.

### Precision diagnostics: expansion without full integration

For rare disease diagnostics, South Korea and Singapore demonstrate the highest level of integration through national programs and insurance coverage.^64-66^ Thailand and China are expanding access, although services remain concentrated in tertiary centers or economically advanced regions.^67-69^ Malaysia and Indonesia are transitioning from research-led access towards early clinical integration, with a national PM strategy in progress.^45,85^ In oncology, China leads in scale through commercially driven expansion aligned with national policy priorities,^42,72^ while South Korea and Singapore demonstrate more structured but selective integration.^73^ Across NGS applications, technological adoption has generally outpaced reimbursement and system-level integration.

### Pharmacogenomics: evidence, not technology, as the constraint

Pharmacogenomic implementation follows a distinct pattern. Thailand leads regionally with UHC reimbursement for selected drug-gene pairs,^8,76,77^ while China shows broad but uneven uptake across specialties.^75^ Singapore demonstrates targeted implementation through pilot programs,^89^ and Malaysia and Indonesia remain early-stage.^90,91^ Here, the limiting factor is not infrastructure but insufficient clinical and economic evidence to support routine reimbursement and standardization.

### AI-enabled PM: early but uneven adoption

AI-enabled PM remains at an early stage across the region but is most advanced in China and South Korea, where national platforms and hospital systems are already embedded into clinical workflows.^80,92,93^ Singapore demonstrates strong research-driven integration,^94,95^ while other countries remain at pilot stages. This suggests that AI-enabled PM adoption depends heavily on broader digital infrastructure and governance readiness.

### Awareness as a cross-cutting consideration

Awareness varies widely and continues to shape both access and implementation. HBOC awareness is highest in Singapore and South Korea due to structured education and advocacy,^24,50^ while elsewhere it remains uneven and largely specialist-driven.^74,96^ Awareness of FH remains low across all settings despite pilot programs,^88,97-99^ and NGS awareness is largely confined to specialist centers outside Korea and China.^100-102^ Pharmacogenomics awareness is more advanced in Thailand and Singapore,^74,89^ while AI-PM awareness remains at an early stage across all countries.^103,104^ Overall, limited awareness and workforce capacity continue to constrain uptake even where technologies are available.

### Stakeholder perspectives

Stakeholder feedback largely reinforced the landscape review, highlighting reimbursement, workforce capacity, affordability, awareness, and regulatory clarity as key implementation considerations.

### Evidence synthesis

According to the PM Implementation Triad, public health genomic screening programs show the highest implementation readiness due to strong alignment across governance, financing, and delivery systems. Precision oncology has relatively high readiness but is constrained by reimbursement and sustainable financing. Rare disease diagnostics depend mainly on infrastructure capacity, whereas pharmacogenomics and AI-enabled precision health platforms remain at early implementation stages.

Country-level Triad analysis identifies four readiness profiles, each associated with distinct implementation priorities. The most ready systems, represented by South Korea and Singapore, demonstrated strong alignment across governance, financing, and infrastructure, and are well positioned to broaden reimbursement and integrate PM more routinely into clinical care. A second group, represented by China, showed advanced governance and infrastructure capacity but weaker financing integration, suggesting that strengthening reimbursement pathways and reducing regional inequities may yield the greatest implementation gains. A third group, represented by Thailand, demonstrated growing system-level readiness through strengthening governance, public financing, and infrastructure, indicating that continued investment in coordinated national implementation and HTA-informed reimbursement could accelerate scale-up. A fourth group comprising Malaysia and Indonesia had established national strategies and basic infrastructure, with implementation driven mainly by pilot programs and selected referral centers, highlighting the need to strengthen infrastructure and workforce capacity before broader implementation.^105^

## Discussion

This study extends previous assessments of PM readiness by examining how financing, governance, infrastructure, and implementation interact across six countries and multiple PM applications. Although technological capacity has expanded substantially across the region, implementation remains uneven. A recurring finding was the disconnect between technology availability and routine clinical integration, suggesting that adoption is constrained less by technical feasibility than by health-system readiness. Across the six countries, broader PM integration was generally observed where these domains were aligned, whereas gaps in any one domain appeared to limit implementation despite technological availability. The Triad synthesis further highlighted a persistent disconnect between technological expansion and financing integration. Several countries have rapidly expanded genomic testing capacity, AI-enabled platforms, and sequencing programs, yet reimbursement pathways, workforce capacity, and implementation frameworks have not progressed at the same pace. This suggests that expanding technological availability alone is insufficient for sustainable PM adoption without parallel investments in financing, governance, and service delivery.

These findings suggest that implementation priorities should be tailored according to both technology type and health-system readiness rather than applying a uniform implementation strategy. More mature systems may benefit from expanding reimbursement, generating real-world evidence, and integrating emerging technologies into routine care, whereas countries in earlier stages of implementation may achieve greater impact by strengthening coordinated governance, sustainable financing, and enabling infrastructure that support clinical workflows, workforce capability, patient awareness, and shared decision-making. Regional collaboration through shared HTA methodologies, multi-country evidence generation, and workforce development may further accelerate implementation across diverse health-system contexts.

### Financing and equity

Financing emerged as a key enabler of PM implementation. Published economic evaluations have reported favorable cost-effectiveness for selected PM applications, including HBOC, pharmacogenomics-guided prescribing, and some NGS-based diagnostic approaches.^38,89,96^ However, economic evidence remains uneven across many technologies and countries, highlighting the need for locally relevant evidence to inform reimbursement decisions. Countries with more established reimbursement pathways generally demonstrated broader integration of PM into routine care, while reliance on self-pay, institutional budgets, or research funding was associated with more fragmented access. However, reimbursement should not be interpreted as an independent causal driver of adoption, as financing is closely intertwined with governance, infrastructure, and broader health-system capacity.

The findings also raise important equity challenges. The concentration of PM services within tertiary centers, coupled with reliance on self-pay financing, may exacerbate existing geographic and socioeconomic disparities in access. Without deliberate efforts to expand reimbursement coverage, strengthen workforce capacity, and improve service integration, the benefits of PM may remain concentrated among populations with greater financial resources and access to specialist services.

### Infrastructural capacity, awareness, and uptake

Infrastructural capacity and digital integration emerged as critical enablers of implementation maturity. Countries with stronger digital interoperability, laboratory capacity, and workforce readiness generally demonstrated broader integration of PM into routine care. However, technology acquisition alone was insufficient; sustainable implementation also depended on robust data governance, workforce development, and effective clinical integration.

Awareness remained an important barrier to PM uptake. While awareness was generally higher for HBOC testing, knowledge of FH screening, pharmacogenomics, and emerging PM technologies remained limited outside specialist settings. These findings suggest that reimbursement and technological availability alone are insufficient to achieve widespread adoption. Clinician education, public engagement, and integration into routine care pathways are equally important for translating PM capacity into sustained clinical use.

### Collaborative efforts of key stakeholder strengthen governance and strategic planning

Governance emerged as a key enabling domain within the PM Implementation Triad. Countries with clearer national strategies, regulatory frameworks, sustained investment in PM research, digital integration, and coordinated implementation efforts generally demonstrated more mature PM integration than those relying primarily on pilot programs or fragmented initiatives.^64,65,78,80,94,102^ These findings suggest that strong governance and cross-sector coordination are important for translating technological capacity into system-wide implementation.

Across countries, recurring challenges include limited real-world evidence for HTA, workforce shortages,^83,106,107^ fragmented digital systems,^32,43,108^ inequitable access,^39,55,109^ and limited local cost-effectiveness data.^38,89^ Addressing these requires coordinated system-level implementation, strengthened HTA capacity, investment in infrastructure and workforce, and integration into clinical pathways. Regional collaborations such as PHAT-TAI may help address these gaps through knowledge sharing, capacity building, and evidence generation across health systems.

While this review identified common implementation patterns across countries, it did not assess how different stakeholders prioritize these barriers or perceive their relative importance. Future studies within the PHAT-TAI program will incorporate stakeholder interviews to further explore reimbursement barriers and enablers, particularly those relating to equity, Delphi consensus methods to identify priority areas, and DEMATEL analyses to examine the interrelationships among implementation factors. Together, these approaches will help validate and prioritize findings of this review and inform more targeted policy strategies for PM implementation in Asia.

### Limitations

This study has several limitations. As a landscape review, findings depended on the availability of published and grey literature and may overrepresent countries with stronger research output or HTA capacity. Consistent with PRISMA-ScR guidance, no formal critical appraisal was conducted; findings should therefore be interpreted as a synthesis of available evidence rather than a weighted assessment of evidence quality. Stakeholder consultations were intended to provide contextual insights and should be viewed as complementary to, rather than representative of, stakeholder perspectives.

While the selected countries reflect a range of health-system contexts and levels of PM implementation maturity, findings may not be generalizable to other Asian settings, in particular, other South Asian or Southeast Asian systems requiring further investigation and stakeholder feedback. In addition, some initiatives may have evolved since data collection. Evidence relating to AI-enabled PM was comparatively limited and relied more heavily on policy reports and program announcements than established genomic applications. Finally, issues relating to genetic discrimination, insurability, cost-effectiveness, and equity were not examined in depth and warrant further investigation. Nevertheless, the integration of literature synthesis with stakeholder perspectives provides a comprehensive overview of PM implementation across diverse Asian health systems.

## Conclusions

PM implementation across Asia remains uneven, reflecting differences in financing, governance, digital infrastructure, and workforce readiness. Countries with stronger alignment across these domains generally demonstrated more mature integration of PM into routine care. Rather than a uniform implementation strategy, countries should prioritize interventions according to their health-system readiness and the requirements of different PM applications. Achieving sustainable scale-up will require coordinated governance, flexible and sustainable financing, infrastructure that supports clinical integration, real-world evidence generation, and regional collaboration to accelerate learning and implementation across diverse health-system contexts.

## Supplementary Material

qxag238_Supplementary_Data

## Data Availability

No new data were created or analyzed in this study. Data sharing is not applicable.

## Declarations

No generative AI tools were used for data generation, analysis, or interpretation in this study.
