# Supplementary material for: Financing and health system capacity for precision medicine in Asia: a six country landscape analysis
Source: Health Aff Sch. 2026 Sep 1;4(9):qxag238. doi: 10.1093/haschl/qxag238 (PMC13564229; doi:10.1093/haschl/qxag238)
Supplement: Supplementary file 1 [file qxag238_supplementary_data.zip › Supplementary File (Final) (1).docx]

**SUPPLEMENTARY FILE**

1. **Supplementary Tables**

**Supplementary Table 1: Precision Medicine Maturity Classification**

| Implementation Maturity | |
| --- | --- |
| Strong | National implementation with routine clinical use, established reimbursement mechanisms, and integration into healthcare pathways. |
| Moderate | Available across multiple centers with partial reimbursement and growing integration into routine care. |
| Emerging | Limited implementation through pilot programs, tertiary centers, or research initiatives with restricted reimbursement. |
| Minimal | Research-based or highly limited availability with little routine clinical integration. |
| Awareness | |
| High | Broad awareness among clinicians and patients supported by national programs, guidelines, or advocacy initiatives. |
| Moderate | Awareness present among specialists but variable among the wider healthcare workforce or public. |
| Low | Limited awareness outside specialized centers or pilot programs. |

**Supplementary Table 2: Initiating Stakeholders of PM in Asian Countries**

| Country | Public health risk screening programs: HBOC Genetic Testing | Public health risk screening programs: Familial Hypercholesterolemia (FH) Cascade Screening | Precision diagnostic and companion diagnostic: NGS (Rare Disease Diagnosis, Medical Oncology, Pharmacogenomics) | AI-integrated precision health system: AI-enabled Precision Medicine |
| --- | --- | --- | --- | --- |
| Singapore | MOH, Agency for Care Effectiveness (ACE), public hospitals, cancer genetics services, academic centers, and advocacy groups | Lipid clinics, public hospitals, and MOH-supported programs | MOH, A*STAR/Genome Institute of Singapore (GIS), public hospitals, and academic centers | Hospital innovation units, A*STAR, technology partners, and national genomics programs |
| Malaysia | MOH hospitals, Ministry of Higher Education teaching hospitals, academic genetics units, and private laboratories | Academic centers and clinical champions | Ministry of Science, Technology and Innovation (MOSTI), academic genomics laboratories, and private laboratories | Academic–industry collaborations |
| Thailand | Ministry of Public Health (MOPH), National Health Security Office (NHSO), academic hospitals, and HITAP | Academic hospitals and specialist clinics | MOPH, NHSO, HITAP, academic hospitals, and national pharmacogenomics working groups | Genomics Thailand Initiative and academic centers |
| Indonesia | University hospitals and private laboratories | Academic centers and clinical champions | National research institutes, university hospitals, and industry partners | MOH innovation programs, university centers, and local technology developers |
| China | Tertiary hospitals and provincial health systems | Specialist centers | Tertiary hospitals, national research institutes, provincial genomics laboratories, and the National Medical Products Administration (NMPA) / Center for Drug Evaluation (CDE) | Hospital-led initiatives and national research programs |
| South Korea | Ministry of Health and Welfare (MOHW), National Cancer Center, major hospital systems, and genetics clinics | Lipid clinics, specialist centers, and research consortia | MOHW, National Health Insurance (NHI), National Cancer Center, hospital consortia, and academic centers | National research consortia and hospital systems |

**Supplementary Table 3: Financing Mechanism of PM in Asian Countries**

| **Country** | **Financing Mechanism** | | | |
| --- | --- | --- | --- | --- |
|  | **Public health risk screening programs: HBOC Genetic Testing** | **Public health risk screening programs: Familial Hypercholesterolemia (FH) Cascade Screening** | **Precision diagnostic and companion diagnostic: NGS (Rare Disease Diagnosis, Medical Oncology, Pharmacogenomics)** | **AI-integrated precision health system: AI-enabled Precision Medicine** |
| **Singapore** | Upcoming government subsidies in Dec 2026. | Partially subsidised (up to ~70%) through public schemes; not universally reimbursed. | Mixed funding through institutional budgets, research grants, philanthropy, and hybrid research–clinical pathways; tumour NGS supported institutionally; MCED largely research-funded. | Primarily research- and institution-funded pilots; no routine reimbursement. |
| **Malaysia** | Mostly out-of-pocket; limited MOH subsidies. | Minimal public funding; largely out-of-pocket or pilot-based. | Mainly out-of-pocket; rare disease testing often supported by NGOs, zakat, and donations; limited private insurance coverage. | Research- or pilot-funded; no dedicated public reimbursement. |
| **Thailand** | Reimbursed for high-risk individuals under UHC and CSMBS. | Limited public funding; early-stage cascade programs. | Selected NGS applications supported under bundled rare-disease packages; pharmacogenomics markers reimbursed; other services require out-of-pocket payment | Government-supported pilots linked to national digital health initiatives; not routinely reimbursed. |
| **Indonesia** | Mostly out-of-pocket, concentrated in private sector. | Small grants and pilot funding; no routine reimbursement. | Mainly research-funded through industry partnerships and pilot programs. | Predominantly research- or innovation-funded pilots. |
| **China** | Mainly out-of-pocket; limited municipal-level reimbursement. | Genetic testing primarily linked to PCSK9 eligibility; otherwise out-of-pocket. | Most NGS services paid out-of-pocket; hospitals often absorb costs due to lack of national billing codes; commercial insurance increasingly relevant. | Institution- or research-funded; uneven adoption across regions. |
| **South Korea** | Reimbursed under NHI. | Cascade testing for relatives not covered; high drug costs limit uptake. | Tumour NGS reimbursed for cancer care; rare disease NGS largely out-of-pocket; pharmacogenomics selectively funded. | Mainly research-funded pilots; no routine reimbursement. |

**Supplementary Table 4: Health Information Systems of PM in Asian Countries**

| **Country** | **Health Information Systems** | | | |
| --- | --- | --- | --- | --- |
|  | **Public health risk screening programs: HBOC Genetic Testing** | **Public health risk screening programs: Familial Hypercholesterolemia (FH) Cascade Screening** | **Precision diagnostic and companion diagnostic: NGS (Rare Disease Diagnosis, Medical Oncology, Pharmacogenomics)** | **AI-integrated precision health system: AI-enabled Precision Medicine** |
| **Singapore** | Genetic results captured within advanced EMR systems across public hospitals. | FH-related genetic and clinical data integrated within EMR and specialist clinic systems. | NGS results routinely captured in EMR and linked to national genomic databases (A*STAR/GIS). | AI dashboards and genomic decision-support tools linked to EMR within selected hospital systems. |
| **Malaysia** | Variable EMR maturity across MOH hospitals; academic centers maintain more robust systems. | Limited digital capture; FH data often recorded outside standardised EMR workflows. | NGS data primarily managed within academic and research databases; MyGenome developing national variant databases with limited clinical integration. | Minimal integration of AI tools into routine EMR; mainly research-based. |
| **Thailand** | Genetic results captured using ICD-10 and scheme-specific billing codes within EMR. | FH-related data captured inconsistently across institutions. | NGS and pharmacogenomic results captured in EMR; interoperability remains incomplete. | Early AI integration within national digital health initiatives; data governance and privacy considerations emerging. |
| **Indonesia** | Partial EMR adoption; genetic results inconsistently recorded. | Limited digital capture beyond tertiary centers. | NGS data primarily stored in research databases; limited EHR–genomics integration pilots underway. | AI data largely confined to pilot or research platforms. |
| **China** | Robust EMR systems in tertiary hospitals capture HBOC and oncology-related genomic data. | FH data captured mainly in specialist centers. | Genomics–EMR integration established in high-tier hospitals; regional cancer and rare disease databases active but fragmented; limited digital capacity at county level. | AI tools integrated within hospital systems in advanced centers, uneven regional deployment. |
| **South Korea** | High EMR penetration enables routine capture of genetic testing results. | FH-related data captured within specialist clinic and claims systems. | NGS and pharmacogenomic data integrated into tertiary hospital EMR and national claims databases. | AI-enabled tools piloted within tertiary hospitals; privacy and governance concerns remain. |

**Supplementary Table 5: Monitoring Framework of PM in Asian Countries**

| **Country** | **Monitoring Framework** | | | |
| --- | --- | --- | --- | --- |
|  | **Public health risk screening programs: HBOC Genetic Testing** | **Public health risk screening programs: Familial Hypercholesterolemia (FH) Cascade Screening** | **Precision diagnostic and companion diagnostic: NGS (Rare Disease Diagnosis, Medical Oncology, Pharmacogenomics)** | **AI-integrated precision health system: AI-enabled Precision Medicine** |
| **Singapore** | ACE conducts HTA reviews; public hospitals maintain clinical registries. | FH dashboards and program-level monitoring implemented through specialist clinics. | Hospital NGS registries track utilisation and outcomes; genomic data integration supported through NGEMR. | AI pilots evaluated through institutional governance frameworks; monitoring embedded within hospital innovation programs. |
| **Malaysia** | Limited national monitoring; academic hospitals maintain internal records. | Minimal structured monitoring; program-level tracking uncommon. | Monitoring remains fragmented across academic centers; MyGenome supports variant databases, but national outcome tracking is limited. | Limited formal monitoring; AI projects tracked mainly within research or pilot settings. |
| **Thailand** | NHSO uses ICD-10 and billing codes to track reimbursed HBOC services. | Early monitoring through specialist and pilot programs. | NHSO and HITAP monitor reimbursed PGx and selected NGS services; national pilots generate implementation and outcomes data. | Pilot-level monitoring linked to national digital health and genomics initiatives. |
| **Indonesia** | Monitoring largely research-led within pilot studies. | Pilot-level registries maintained by academic centers. | Research registries track NGS use, with limited integration beyond tertiary hospitals. | AI monitoring confined to pilot evaluations in selected centers. |
| **China** | Monitoring conducted through hospital information systems and provincial registries. | Limited routine monitoring; often linked to treatment eligibility audits. | Oncology NGS, MRD/cell-free DNA, and rare disease genomics are monitored through hospital and provincial platforms; inconsistent coding limits HTA use. | AI-enabled monitoring piloted within large tertiary hospitals; regional variability remains high. |
| **South Korea** | Cancer genomics outcomes monitored through national platforms. | Limited routine cascade monitoring. | National platforms (e.g. K-MASTER, KOSMOS) support longitudinal tracking of tumour genomics and treatment outcomes; PGx monitored through observational programs. | AI-linked monitoring piloted in major tertiary hospitals and national research programs |

**Supplementary Table 6: Governance and Financing Structures by Country**

| Country | Key Decision-Maker | Role |
| --- | --- | --- |
| **Singapore** | MOH; ACE; National Precision Medicine Program; PRECISE, Public healthcare clusters | Centralised system: MOH sets policy and financing; ACE^1^ conducts HTA to inform funding and guidelines; National Precision Medicine drives national genomics strategy (e.g. SG100K); PRECISE supports clinical implementation; public clusters deliver PM within an integrated system supported by MediSave, MediShield Life, and NGEMR |
| **Malaysia** | MOH; MyGenome; MaHTAS; Public hospitals; Private sector | More fragmented public–private system: MOH oversees policy and public delivery; MyGenome supports genomics infrastructure; MaHTAS conducts HTA; PM implementation is largely pilot-driven in public hospitals, with additional private sector provision mainly out-of-pocket |
| **Thailand** | MOPH; NHSO; CSMBS; Social Security Office; HITAP | Multi-payer system: MOPH sets policy; NHSO manages Universal Coverage Scheme (UCS) for the majority; CSMBS and Social Security Office provide parallel schemes; HITAP conducts HTA to inform coverage and reimbursement decisions for PM technologies |
| **Indonesia** | MOH; BPJS Kesehatan; Genomic Sovereignty Initiative (GSI); Academic centers; Public hospitals | Decentralised system: MOH sets policy; BPJS manages national insurance (JKN); GSI supports genomics capacity; PM implementation is largely research- and pilot-driven with limited integration into routine care |
| **China** | National Health Commission (NHC); National Healthcare Security Administration (NHSA); NMPA; Provincial authorities; Public tertiary hospitals | Mixed system: NHC sets policy; NHSA governs reimbursement; NMPA regulates PM technologies; provinces oversee implementation; tertiary hospitals drive PM adoption, resulting in regional variation |
| **South Korea** | MOHW; National Health Insurance Service (NHIS); Health Insurance Review and Assessment Service (HIRA); K-MASTER; KOSMOS; Korean National DNA Program (K-DNA) | Centralised system: MOHW sets policy; NHIS provides national coverage; HIRA conducts HTA; national programs support genomic data and precision oncology integration into routine care |

**Supplementary Table 7: Healthcare Integration of PM in Asian Countries**

| **Country** | **Healthcare Integration** | | | |
| --- | --- | --- | --- | --- |
|  | **Public health risk screening programs: HBOC Genetic Testing** | **Public health risk screening programs: Familial Hypercholesterolemia (FH) Cascade Screening** | **Precision diagnostic and companion diagnostic: NGS (Rare Disease Diagnosis, Medical Oncology, Pharmacogenomics)** | **AI-integrated precision health system: AI-enabled Precision Medicine** |
| **Singapore** | Integrated into oncology pathways with established referral networks. | Integrated within cardiology and lipid clinics; structured referral pathways present. | Embedded in multidisciplinary tumour boards and rare disease workflows across public hospitals. | AI decision-support tools piloted within clinical workflows; not yet routine. |
| **Malaysia** | Services largely concentrated in tertiary centers; limited access beyond academic hospitals. | Minimal integration; limited specialist capacity. | NGS largely operates on a research-to-service model with limited system-wide integration. | Limited clinical integration; pilots remain research-led. |
| **Thailand** | Integrated into oncology care pathways; downstream care not uniformly standardised. | Early-stage integration; variable across institutions. | NGS available in tertiary centers for oncology and rare diseases; uneven access outside Bangkok. | Limited clinical integration; early pilots ongoing. |
| **Indonesia** | Primarily delivered through tertiary and private hospitals; limited national integration. | Mostly academic or pilot-based. | Hybrid research–clinical use of NGS in selected centers; limited referral pathways. | Early-stage AI pilots with minimal routine clinical integration. |
| **China** | Strong integration in tertiary oncology centers for targeted therapy. | Limited routine integration; referral-dependent. | NGS embedded in oncology workflows at tertiary hospitals; rare disease genomics relies on referral networks due to uneven expertise. | AI tools used operationally within hospitals; not formally reimbursed or standardised. |
| **South Korea** | Integrated into tertiary genetics and oncology clinics. | Integrated within specialist lipid clinics; cascade coverage limited. | Tumour NGS embedded in oncology workflows; rare disease NGS largely confined to major hospitals. | AI tools remain largely research- or pilot-based. |

**Supplementary Table 8 : Access for PM Types Across Countries**

| Country | | HBOC Genetic Testing | FH Cascade Screening | NGS: Rare Disease Diagnosis | NGS: Medical Oncology | NGS: Pharmacogenomics | AI-enabled PM |
| --- | --- | --- | --- | --- | --- | --- | --- |
| **Singapore** | Available through public hospital cancer genetics services; partially subsidised | | National FH programs with subsidised access through lipid clinics and genetic assessment services | Available through national and hospital programs (e.g., BRIDGES) | Routine tumour NGS in tertiary care; liquid biopsy and MCED mainly research-based | Available in selected tertiary settings; limited routine reimbursement | Pilot implementation for genomic interpretation and clinical decision support |
| **Malaysia** | Available through university hospitals and Cancer Research Malaysia; largely self-pay | | Academic-led pilots; routine cascade screening not established | Available in tertiary and university hospitals; largely research-based | Available mainly through private providers; limited public-sector adoption | Limited use in selected centers | Early-stage pilots in tertiary centers |
| **Thailand** | Reimbursed for high-risk individuals under UHC | | Pilot programs; no nationwide cascade screening | Available in tertiary centers; regional disparities remain | Available in tertiary centers with expanding access | UHC reimbursement for selected drug–gene pairs | Early clinical pilots in major centers |
| **Indonesia** | Available mainly through private laboratories and selected urban hospitals; largely self-pay | | Academic pilots only; no national programs | Primarily research-based through national initiatives and partnerships | Emerging access through selected collaborations and tertiary centers | Limited pilot implementation | Pilot programs under national digital health initiatives |
| **China** | Widely available in tertiary hospitals; selective municipal reimbursement | | Limited availability; often linked to PCSK9 inhibitor eligibility | Expanding through national and provincial initiatives; concentrated in major centers | Widely available in tertiary oncology centers; MCED largely research-based | Expanding across multiple specialties with regional variation | Widely deployed in tertiary centers; reimbursement varies by region |
| **South Korea** | Reimbursed under NHI for probands; access varies by center | | Registry-supported programs with partial reimbursement | Available through national rare disease programs and NHI coverage | Widely available under NHI reimbursement | Available across multiple specialties; selective reimbursement | National platform-supported pilots; not yet routine |

**Supplementary Table 9: Awareness for PM Types Across Countries**

| Country | HBOC Genetic Testing | FH Cascade Screening | NGS: Rare Disease Diagnosis | NGS: Medical Oncology | NGS: Pharmacogenomics | AI-enabled PM |
| --- | --- | --- | --- | --- | --- | --- |
| **Singapore** | High awareness through hereditary cancer clinics, advocacy groups, and national screening initiatives | Moderate awareness through advocacy campaigns, lipid clinics, and professional education | Awareness largely confined to genetics specialists and rare disease programs | Moderate awareness in tertiary centers; public awareness developing | Limited awareness outside specialist settings | Growing professional awareness through oncology and genomics pilots |
| **Malaysia** | Moderate awareness through Cancer Research Malaysia and advocacy initiatives | Low awareness outside specialist settings | Limited awareness among genetics specialists and academic centers | Low awareness outside research and private-sector settings | Limited awareness among clinicians | Early professional awareness through national precision medicine initiatives |
| **Thailand** | Moderate awareness supported by UHC coverage and breast cancer campaigns | Low awareness outside major centers | Specialist-driven awareness through rare disease programs | Limited public awareness; concentrated in tertiary centers | Increasing clinician awareness through national PGx initiatives | Growing awareness among oncology clinicians and researchers |
| **Indonesia** | Low awareness outside major urban centers | Low awareness and advocacy | Awareness largely confined to research communities | Limited awareness outside research settings | Minimal awareness beyond specialist institutes | Early awareness through national digital health initiatives |
| **China** | Increasing awareness in urban tertiary centers; lower awareness in rural areas | Low awareness among clinicians and the public | Growing awareness through genomics initiatives | Urban-centered awareness driven by commercial and research programs | Awareness concentrated among specialists | High clinician awareness in leading tertiary hospitals |
| **South Korea** | High awareness through hereditary cancer clinics and patient advocacy groups | Moderate clinician awareness; limited public awareness | Strong awareness through national rare disease programs | Moderate awareness within oncology and research settings | Increasing clinician awareness through PGx programs | Strong clinician awareness through national AI initiatives |

**Supplementary Table 10: Implementation of PM Types Across Countries**

| Country | HBOC Genetic Testing | FH Cascade Screening | NGS: Rare Disease Diagnosis | NGS: Medical Oncology | NGS: Pharmacogenomics | AI-enabled PM |
| --- | --- | --- | --- | --- | --- | --- |
| **Singapore** | Routine implementation in public hospitals | Pilot-to-program implementation | Research-enabled clinical implementation | Routine tumour NGS; MCED largely research-based | Selective implementation | Advanced pilot implementation |
| **Malaysia** | Limited tertiary-center implementation | Pilot implementation | Small-scale implementation | Limited tertiary-center implementation | Early-stage implementation | Early-stage implementation |
| **Thailand** | Routine implementation for high-risk groups | Pilot implementation | Expanding implementation in tertiary centers | Moderate implementation | Strong implementation | Early clinical implementation |
| **Indonesia** | Limited implementation | Pilot implementation | Research-led implementation | Research-led implementation | Pilot implementation | Early-stage implementation |
| **China** | Broad tertiary-center implementation | Limited implementation | Strong implementation in major center | Strong tertiary- center implementation | Expanding implementation | Rapid implementation in leading centers |
| **South Korea** | Routine implementation | Moderate implementation | Strong implementation | Strong implementation | Moderate implementation | Advanced implementation |

**Supplementary Table 11: Routine Practice Availability of PM in Asian Countries**

| **Country** | **Routine Practice Availability** | | | |
| --- | --- | --- | --- | --- |
|  | **Public health risk screening programs: HBOC Genetic Testing** | **Public health risk screening programs: Familial Hypercholesterolemia (FH) Cascade Screening** | **Precision diagnostic and companion diagnostic: NGS (Rare Disease Diagnosis, Medical Oncology, Pharmacogenomics)** | **AI-integrated precision health system: AI-enabled Precision Medicine** |
| **Singapore** | Routinely offered in public hospitals; partially subsidised; not universally reimbursed. | Cascade screening available in specialist clinics; partially subsidised (e.g. Medisave), not population-wide. | **Rare disease:** Available in national and hospital programs.  **Medical oncology:** Tumour NGS widely used for treatment selection; MCED remains research-based.  **Pharmacogenomics:** Available in tertiary centers and selected drug–gene pathways; limited routine reimbursement. | Early AI deployment linked to national genomics initiatives (SG100K, National Precision Medicine Initiative); pilots in clinical decision support and genomics interpretation. |
| **Malaysia** | Available in tertiary centers; limited by quotas and funding. | Inconsistently implemented; largely pilot-based. | **Rare disease:** NGS largely research-driven; reliance on NGOs, zakat, and fundraising.  **Medical oncology:** Tumour NGS mainly in academic centers; MCED largely private.  **Pharmacogenomics:** Limited, non-routine use. | Limited routine AI integration; largely exploratory or research-based. |
| **Thailand** | Routinely reimbursed under UHC and CSMBS. | Early-stage cascade screening programs; not yet nationwide. | **Rare disease:** NGS available in tertiary centers.  **Medical oncology:** Tumour NGS available; geographic disparities persist.  **Pharmacogenomics:** **Widely implemented and reimbursed** under UHC (e.g. HLA-B15:02, HLA-B57:01). | AI-assisted tools piloted in selected centers; not system-wide. |
| **Indonesia** | Available mainly in private laboratories and select urban hospitals. | Limited to pilot programs. | **Rare disease:** NGS supported through national initiatives and industry partnerships; research-heavy.  **Medical oncology:** Limited routine use.  **Pharmacogenomics:** Minimal, pilot-based. | Early-stage AI pilots in selected centers only. |
| **China** | Widely available in tertiary hospitals. | Not routine; primarily linked to PCSK9 eligibility. | **Rare disease:** Increasing use in major centers.  **Medical oncology:** NGS widely used for treatment selection, MRD/cell-free DNA; **strong MCED research activity,** limited routine adoption.  **Pharmacogenomics:** Available in major hospitals; regional variability. | AI tools increasingly embedded within hospital systems; uneven access across provinces. |
| **South Korea** | Routinely offered and **NHI-reimbursed.** | Present but constrained by high lipid-lowering drug costs. | **Rare disease:** Limited reimbursement coverage.  **Medical oncology:** **Tumour NGS reimbursed under NHI** for cancer pathology and treatment selection; MCED research-only.  **Pharmacogenomics:** Available in selected pathways; not universally reimbursed. | AI-assisted precision medicine largely pilot-based, supported by national platforms (e.g. K-MASTER, KOSMOS). |

2. **Abbreviations**

| **Acronym** | **Description** |
| --- | --- |
| ACE | Agency for Care Effectiveness |
| AI | Artificial Intelligence |
| **AI-PM** | Artificial Intelligence–enabled precision medicine |
| AMR | Antimicrobial Resistance |
| A*STAR | Agency for Science, Technology and Research (Singapore) |
| BRCA | Breast Cancer Gene (*BRCA* 1/2) |
| BRIDGES | Bringing Research Innovations for the Diagnosis of GEnetic diseases in Singapore (Singapore) |
| BREATHE | BREAst screening Tailored for HEr (Singapore breast cancer screening program) |
| CDE | Center for Drug Evaluation (China) |
| CSMBS | Civil Servant Medical Benefit Scheme (Thailand) |
| cfDNA | Cell-free DNA |
| EHR / EMR | Electronic Health Record / Electronic Medical Record |
| FH | Familial Hypercholesterolemia |
| GIS | Genome Institute of Singapore |
| GP | General practitioner |
| **HBOC** | Hereditary Breast and Ovarian Cancer |
| HITAP | Health Intervention and Technology Assessment Program (Thailand) |
| HTA | Health Technology Assessment |
| ICD-10 | International Classification of Diseases, 10th Revision |
| KDCA | Korea Disease Control and Prevention Agency (South Korea) |
| K-MASTER | Korean Cancer Precision Medicine Diagnosis and Treatment Enterprise |
| KOSMOS | KOrean precision Medicine Networking Group Study of MOlecular profiling-guided therapy based on genomic alterations in advanced solid tumors (Korea) |
| MaHTAS | Malaysian Health Technology Assessment Section |
| MCED | Multi-cancer early detection |
| **MOH** | Ministry of Health |
| **MOHW** | Ministry of Health and Welfare (South Korea) |
| MOPH | Ministry of Public Health of Thailand |
| MOSTI | Ministry of Science, Technology and Innovation (Malaysia) |
| MRD | Molecular Residual Disease |
| **NCCS** | National Cancer Center Singapore |
| NCIS | National University Cancer Institute, Singapore |
| NECA | National Evidence-based Healthcare Collaborating Agency (South Korea) |
| NGEMR | Next Generation Electronic Medical Record (Singapore) |
| NGS | Next-generation sequencing |
| NHI | National Health Insurance (South Korea) |
| **NHSO** | National Health Security Office (Thailand) |
| NICE | National Institute for Health and Care Excellence (United Kingdom) |
| NMPA | National Medical Products Administration (China) |
| NSTDA | National Science and Technology Development Agency (Thailand) |
| OOP | Out-of-pocket payment |
| PCSK9 | Proprotein convertase subtilisin/kexin type 9 |
| PGx | Pharmacogenomics |
| PHAT-TAI | HTAsiaLink Precision HeAlth Transformative Technology Assessment Initiative |
| PIRO | Population, Intervention, Reimbursement, Outcome framework |
| PM | Precision medicine |
| **PRISMA** | Preferred Reporting Items for Systematic Reviews and Meta-Analyses |
| PRECISE | National Precision Medicine Program (Singapore) |
| RapidSeq | Rapid genomic sequencing program (Singapore) |
| **SG100K** | Singapore 100,000 Genomes Project |
| SPOT-MAS | Screening for the Presence Of Tumor by Methylation And Size |
| **TCELS** | Thailand Center of Excellence for Life Sciences |
| UHC | Universal Health Coverage (Thailand) |
| **WES** | Whole-exome sequencing |

3. **Search Strategy**

PUBMED

|  | **Search Terms** | **Articles** |
| --- | --- | --- |
| #1 | ("precision medicine"[tiab] OR "personalized medicine"[tiab] OR "genomic medicine"[tiab] OR "genetic testing"[tiab] OR "HBOC"[tiab] OR “HBOC testing"[tiab] OR "BRCA"[tiab] OR "BRCA testing"[tiab] OR "familial hypercholesterolemia"[tiab] OR "cascade screening"[tiab] OR "next generation sequencing"[tiab] OR "Rare Disease Diagnosis"[tiab] OR "cancer risk screening"[tiab] OR “medical oncology” [tiab] OR "newborn screening" [tiab] OR "Companion Diagnostic "[tiab] OR “pharmacogenomics” [tiab] OR (("artificial intelligence"[tiab] OR "machine learning"[tiab] OR "risk score"[tiab]) AND "genomic profiling"[tiab])) | 188,600 |
| #2 | ("reimbursement"[tiab] OR "health financing"[tiab] OR "healthcare financing"[tiab] OR "insurance coverage"[tiab] OR "public financing"[tiab] OR "health technology assessment"[tiab] OR "policy"[tiab] OR "implementation"[tiab] OR "integration"[tiab] OR "health system integration"[tiab]) | 1,037,094 |
| #3 | ("Singapore"[tiab] OR "Malaysia"[tiab] OR "Thailand"[tiab] OR "Indonesia"[tiab] OR "South Korea"[tiab] OR "China"[tiab]) | 501,160 |
| #1 AND #2 AND #3 | ((("precision medicine"[tiab] OR "personalized medicine"[tiab] OR "genomic medicine"[tiab] OR "genetic testing"[tiab] OR “HBOC"[tiab] OR “HBOC testing"[tiab] OR "BRCA"[tiab] OR "BRCA testing"[tiab] OR "familial hypercholesterolemia"[tiab] OR "cascade screening"[tiab] OR "next generation sequencing"[tiab] OR "Rare Disease Diagnosis"[tiab] OR "cancer risk screening"[tiab] OR “medical oncology”[tiab] OR "newborn screening" [tiab] OR "Companion Diagnostic "[tiab] OR “pharmacogenomics” [tiab] OR (("artificial intelligence"[tiab] OR "machine learning"[tiab] OR "risk score"[tiab]) AND "genomic profiling"[tiab]))) AND (("reimbursement"[tiab] OR "health financing"[tiab] OR "healthcare financing"[tiab] OR "insurance coverage"[tiab] OR "public financing"[tiab] OR "health technology assessment"[tiab] OR "policy"[tiab] OR "implementation"[tiab] OR "integration"[tiab] OR "health system integration"[tiab]))) AND (("Singapore"[tiab] OR "Malaysia"[tiab] OR "Thailand"[tiab] OR "Indonesia"[tiab] OR "South Korea"[tiab] OR "China"[tiab])) | **305** |

**WEB OF SCIENCE**

|  | Search Terms | Articles |
| --- | --- | --- |
| #1 (select Topic) | (("precision medicine" OR "personalized medicine" OR "genomic medicine" OR "genetic testing" OR "HBOC" OR "HBOC testing" OR "BRCA" OR "BRCA testing" OR "familial hypercholesterolemia" OR "cascade screening" OR "next generation sequencing" OR "newborn screening" OR "cancer risk screening" OR “medical oncology” OR “Companion Diagnostic” OR ““pharmacogenomics” OR "AI in oncology" OR (("artificial intelligence" OR "machine learning" OR "risk score") AND "genomic profiling"))) | 208,600 |
| #2 (select Topic) | ("reimbursement" OR "health financing" OR "healthcare financing" OR "insurance coverage" OR "public financing" OR "health technology assessment" OR "policy" OR "implementation" OR "integration" OR "health system integration") | 3,549,382 |
| #3 (select Topic) | ("Asia" OR "Singapore" OR "Malaysia" OR "Thailand" OR "Indonesia" OR "South Korea" OR "China") | 1,760,055 |
| #1 AND #2 AND #3 | (("precision medicine" OR "personalized medicine" OR "genomic medicine" OR "genetic testing" OR "HBOC" OR "HBOC testing" OR "BRCA" OR "BRCA testing" OR "familial hypercholesterolemia" OR "cascade screening" OR "next generation sequencing" OR "newborn screening" OR "cancer risk screening" OR “medical oncology” OR “Companion Diagnostic” OR ““pharmacogenomics” OR "AI in oncology" OR (("artificial intelligence" OR "machine learning" OR "risk score") AND "genomic profiling"))) AND ("reimbursement" OR "health financing" OR "healthcare financing" OR "insurance coverage" OR "public financing" OR "health technology assessment" OR "policy" OR "implementation" OR "integration" OR "health system integration") AND ("Asia" OR "Singapore" OR "Malaysia" OR "Thailand" OR "Indonesia" OR "South Korea" OR "China") | **482** |

**SCOPUS**

|  | Search Terms | Articles |
| --- | --- | --- |
| #1 | ("precision medicine" OR "personalized medicine" OR "genomic medicine" OR "genetic testing" OR "HBOC" OR "HBOC testing" OR "BRCA" OR "BRCA testing" OR "familial hypercholesterolemia" OR "cascade screening" OR "next generation sequencing" OR "newborn screening" OR "cancer risk screening" OR “Companion Diagnostics” OR ““pharmacogenomics" OR “AI in oncology" OR ( ( "artificial intelligence" OR "machine learning" OR "risk score" ) AND "genomic profiling" ) | 314,655 |
| #2 | (TITLE-ABS-KEY("reimbursement" OR "health financing" OR "healthcare financing" OR "insurance coverage" OR "public financing" OR "health technology assessment" OR "policy" OR "implementation" OR "integration" OR "health system integration")) | 5,470,618 |
| #3 | (TITLE-ABS-KEY("Asia" OR "Singapore" OR "Malaysia" OR "Thailand" OR "Indonesia" OR "South Korea" OR "China")) | 2,593,217 |
| #1 AND #2 AND #3 | ( ( TITLE-ABS-KEY ( "precision medicine" OR "personalized medicine" OR "genomic medicine" OR "genetic testing" OR "HBOC" OR "HBOC testing" OR "BRCA" OR "BRCA testing" OR "familial hypercholesterolemia" OR "cascade screening" OR "next generation sequencing" OR "newborn screening" OR "cancer risk screening" OR “Companion Diagnostics” OR ““pharmacogenomics" OR "AI in oncology" OR ( ( "artificial intelligence" OR "machine learning" OR "risk score" ) AND "genomic profiling" ) ) ) ) AND ( ( TITLE-ABS-KEY ( "reimbursement" OR "health financing" OR "healthcare financing" OR "insurance coverage" OR "public financing" OR "health technology assessment" OR "policy" OR "implementation" OR "integration" OR "health system integration" ) ) ) AND ( ( TITLE-ABS-KEY ( "Asia" OR "Singapore" OR "Malaysia" OR "Thailand" OR "Indonesia” OR "South Korea" OR "China" ) ) ) | **913** |

**EMBASE**

|  | Search Terms | Articles |
| --- | --- | --- |
| #1 | ('precision medicine'/exp OR 'personalized medicine'/exp OR 'genomic medicine'/exp OR 'genetic testing'/exp OR 'hboc'/exp OR 'hboc testing':ti,ab OR 'brca'/exp OR 'brca testing':ti,ab OR 'familial hypercholesterolemia'/exp OR 'cascade screening':ti,ab OR 'next generation sequencing'/exp OR 'newborn screening'/exp OR 'cancer risk screening':ti,ab OR 'companion diagnostic':ti,ab OR ‘pharmacogenomics’:ti,ab OR 'ai in oncology':ti,ab OR (('artificial intelligence'/exp OR 'machine learning'/exp OR 'risk score':ti,ab) AND 'genomic profiling':ti,ab)) | 539,783 |
| #2 | ('reimbursement'/exp OR 'health financing':ti,ab OR 'healthcare financing':ti,ab OR 'insurance coverage':ti,ab OR 'public financing':ti,ab OR 'health technology assessment'/exp OR 'policy':ti,ab OR 'implementation':ti,ab OR 'integration':ti,ab OR 'health system integration':ti,ab) | 1,329,733 |
| #3 | ('asia'/exp OR 'singapore'/exp OR 'malaysia'/exp OR 'thailand'/exp OR 'indonesia'/exp OR 'south korea'/exp OR 'china'/exp) | 1,615,896 |
| #1 AND #2 AND #3 | #1 AND #2 AND #3 | **1,652** |

4. **Preferred Reporting Items for Systematic reviews and Meta-Analyses extension for Scoping Reviews (PRISMA-ScR) Checklist**

| **SECTION** | **ITEM** | **PRISMA-ScR CHECKLIST ITEM** | **REPORTED ON PAGE #** |
| --- | --- | --- | --- |
| **TITLE** | | | |
| Title | 1 | Identify the report as a scoping review. | 1 to 3  (Title Page) |
| **ABSTRACT** | | | |
| Structured summary | 2 | Provide a structured summary that includes (as applicable): background, objectives, eligibility criteria, sources of evidence, charting methods, results, and conclusions that relate to the review questions and objectives. | 4 to 5 (Abstract) |
| **INTRODUCTION** | | | |
| Rationale | 3 | Describe the rationale for the review in the context of what is already known. Explain why the review questions/objectives lend themselves to a scoping review approach. | 7 to 10 |
| Objectives | 4 | Provide an explicit statement of the questions and objectives being addressed with reference to their key elements (e.g., population or participants, concepts, and context) or other relevant key elements used to conceptualize the review questions and/or objectives. | 10 |
| **METHODS** | | | |
| Protocol and registration | 5 | Indicate whether a review protocol exists; state if and where it can be accessed (e.g., a Web address); and if available, provide registration information, including the registration number. | NA |
| Eligibility criteria | 6 | Specify characteristics of the sources of evidence used as eligibility criteria (e.g., years considered, language, and publication status), and provide a rationale. | 11 to 12 |
| Information sources* | 7 | Describe all information sources in the search (e.g., databases with dates of coverage and contact with authors to identify additional sources), as well as the date the most recent search was executed. | 10 to 13 |
| Search | 8 | Present the full electronic search strategy for at least 1 database, including any limits used, such that it could be repeated. | Supplementary File (13 to 16) |
| Selection of sources of evidence† | 9 | State the process for selecting sources of evidence (i.e., screening and eligibility) included in the scoping review. | 10 to 13 |
| Data charting process‡ | 10 | Describe the methods of charting data from the included sources of evidence (e.g., calibrated forms or forms that have been tested by the team before their use, and whether data charting was done independently or in duplicate) and any processes for obtaining and confirming data from investigators. | 12 to 13 |
| Data items | 11 | List and define all variables for which data were sought and any assumptions and simplifications made. | 12 to 13 |
| Critical appraisal of individual sources of evidence§ | 12 | If done, provide a rationale for conducting a critical appraisal of included sources of evidence; describe the methods used and how this information was used in any data synthesis (if appropriate). | Not done |
| Synthesis of results | 13 | Describe the methods of handling and summarizing the data that were charted. | 13 to 14 |
| **RESULTS** | | | |
| Selection of sources of evidence | 14 | Give numbers of sources of evidence screened, assessed for eligibility, and included in the review, with reasons for exclusions at each stage, ideally using a flow diagram. | 10 to 12,  Figure 1 |
| Characteristics of sources of evidence | 15 | For each source of evidence, present characteristics for which data were charted and provide the citations. | 14 to 24 |
| Critical appraisal within sources of evidence | 16 | If done, present data on critical appraisal of included sources of evidence (see item 12). | Not done |
| Results of individual sources of evidence | 17 | For each included source of evidence, present the relevant data that were charted that relate to the review questions and objectives. | 14 to 24 |
| Synthesis of results | 18 | Summarize and/or present the charting results as they relate to the review questions and objectives. | 23 to 24 |
| **DISCUSSION** | | | |
| Summary of evidence | 19 | Summarize the main results (including an overview of concepts, themes, and types of evidence available), link to the review questions and objectives, and consider the relevance to key groups. | 24 to 29 |
| Limitations | 20 | Discuss the limitations of the scoping review process. | 29 to 30 |
| Conclusions | 21 | Provide a general interpretation of the results with respect to the review questions and objectives, as well as potential implications and/or next steps. | 30 |
| **FUNDING** | | | |
| Funding | 22 | Describe sources of funding for the included sources of evidence, as well as sources of funding for the scoping review. Describe the role of the funders of the scoping review. | 2 to 3  (Title Page) |

JBI = Joanna Briggs Institute; PRISMA-ScR = Preferred Reporting Items for Systematic reviews and Meta-Analyses extension for Scoping Reviews.

* Where *sources of evidence* (see second footnote) are compiled from, such as bibliographic databases, social media platforms, and Web sites.

† A more inclusive/heterogeneous term used to account for the different types of evidence or data sources (e.g., quantitative and/or qualitative research, expert opinion, and policy documents) that may be eligible in a scoping review as opposed to only studies. This is not to be confused with *information sources* (see first footnote).

‡ The frameworks by Arksey and O’Malley (6) and Levac and colleagues (7) and the JBI guidance (4, 5) refer to the process of data extraction in a scoping review as data charting*.*

§ The process of systematically examining research evidence to assess its validity, results, and relevance before using it to inform a decision. This term is used for items 12 and 19 instead of "risk of bias" (which is more applicable to systematic reviews of interventions) to include and acknowledge the various sources of evidence that may be used in a scoping review (e.g., quantitative and/or qualitative research, expert opinion, and policy document).

*From:* Tricco AC, Lillie E, Zarin W, O'Brien KK, Colquhoun H, Levac D, et al. PRISMA Extension for Scoping Reviews (PRISMAScR): Checklist and Explanation. Ann Intern Med. 2018;169:467–473. [doi: 10.7326/M18-0850](http://annals.org/aim/fullarticle/2700389/prisma-extension-scoping-reviews-prisma-scr-checklist-explanation).
